# Overexpression of miR-142-5p inhibits the progression of nonalcoholic steatohepatitis by targeting TSLP and inhibiting JAK-STAT signaling pathway

**DOI:** 10.18632/aging.103172

**Published:** 2020-05-15

**Authors:** Chao Zhou, Pu Wang, Lei Lei, Yi Huang, Yue Wu

**Affiliations:** 1 Department of Gastroenterology, Sichuan Academy of Medical Sciences and Sichuan Provincial People’s Hospital, Chengdu 610072, Sichuan, China; 2 Department of Laboratory Medicine, Sichuan Academy of Medical Sciences and Sichuan Provincial People’s Hospital, Chengdu 610072, Sichuan, China; 3 Personalized Drug Therapy Key Laboratory of Sichuan Province, Department of Pharmacy, Sichuan Academy of Medical Sciences and Sichuan Provincial People’s Hospital, Chengdu 610072, Sichuan, China

**Keywords:** non-alcoholic steatohepatitis, miR142-5p, TSLP, JAK-STAT signaling pathway

## Abstract

This study aimed to figure out the underlying mechanism of miR-142-5p in the non-alcoholic steatohepatitis (NASH). Bioinformatics, luciferase assay and Western blot were performed. The NASH mouse model was established through feeding a high fat diet (HFD). Relative expressions of miR-142-5p, thymic stromal lymphopoietin (*TSLP)*, inflammatory factors were detected by qRT-PCR. The injury level of liver was assessed via measurement of serum alanine aminotransferase (ALT) and serum aspartate aminotransferase (AST). H&E staining and Masson’s trichrome staining examine the liver fatty degeneration and fibrosis. MiR-142-5p and TSLP were differentially expressed and JAK-STAT signaling pathway was activated in the NASH group. Luciferase assay identified that *TSLP* was the downstream target of miR-142-5p. Through overexpression of miR-142-5p, ALT and AST in serum were inhibited, pro-inflammatory factors, liver fatty degeneration and fibrosis in liver tissues were decreased, while anti-inflammatory factors were increased. Overexpression of *TSLP* and JAK-STAT signaling pathway activation could reverse the effects of miR-142-5p on NASH. Taken together, overexpression of miR-142-5p could attenuate NASH progression via inhibiting *TSLP* and JAK-STAT pathway. MiR-142-5p might be a novel latent target for NASH therapy.

## INTRODUCTION

Non-alcoholic steatohepatitis (NASH) is an increasing common chronic liver inflammatory disease characterized by hepatic steatosis and inflammation [1]. It is developed from nonalcoholic fatty liver disease (NAFLD) and can evolve to cirrhosis or hepatocellular carcinoma (HCC) [2]. A current study estimates that about 25% of the world population has NAFLD [3]. Currently, there is no approved drug regimen for NASH [4]. Due to the lack of treatment and the global prevalence of obesity, the prevalence of NAFLD may increase, causing a serious health crisis in the coming decades. A recent research model estimates that NASH-related liver deaths will increase by 178% by 2030 [5]. Because of this, finding new disease damage biomarkers or possible therapeutic targets will become one of the important concerns in this field.

MicroRNAs (miRNAs), the cluster with 18-25 nucleotide RNAs, are one of the subgroups of non-coding RNAs. MiRNAs expression profiles differ between disease states and normal tissue, so it plays an important role in diseases [6]. Increasing reports showed that a large number of miRNAs were abnormally expressed in NASH and suggested that miRNAs could be potential biomarkers and therapeutic targets for NASH [22]. For example, Clarke et al. found that miR-122 were significantly increased in serum in the mouse model of NASH and suggested that miR-122 could be a potential sensitive biomarker for the early detection of hepatotoxicity [23]. Loyer et al. found that miRNA-21 was upregulated in NASH and contributed to the disease by inhibiting the expression of PPARα [24]. Other miRNAs such as miR-33a, miR-34a, and miR-24 also have shown potential status as factors contributing to or involved in the development of NASH/NAFLD or as markers for hepatic inflammation in the progression of NASH/NAFLD [7]. Notably, studies have shown that miR-142-5p could regulate the expression of pro-fibrogenic genes and down-regulation of miR-142-3p might lead to elevated levels of IL-6 in aged mice [8, 9]. In addition, miR-142-5p was shown to be significantly downregulated in HCC [10, 11]. Another discussion on the mechanism of fatty liver also found that lipid-lowering drugs can reduce lipids by up-regulating miR-142-5p [12]. At the same time, it is possible that the development of NASH will progress to HCC [13]. Therefore, miR-142-5p has the potential to become a new target miRNA for the treatment of NASH.

Thymic stromal lymphopoietin (*TSLP*), which is a master regulator of Th2-driven inflammation, has been studied in various allergic diseases such as asthma and inflammatory bowel diseases (IBD). Park et al. reported that *TSLP* was a key mediator in the IBD progression [14]. Maria-Isabel et al. found that *TSLP* could trigger a cell-autonomous dendritic cell migration [15]. These researches suggested that *TSLP* might take an important part in the inflammatory diseases. Furthermore, a previous study has shown that *TSLP* induces the release of CCL2 from fibroblasts via STAT3, which in turn induces monocyte chemotaxis [16]. This study suggests that TSLP may affect the process of fibrosis through longer boots that affect the expression of the STAT3. It is worth noting that STAT3 is an important regulator of JAK-STAT signaling pathway, and a large number of studies have shown that JAK-STAT signaling pathway regulates the occurrence and development of NASH [17–19]. However, there are few studies on TSLP and NASH, and whether its effect on fibrosis can affect NASH is worthy of further study.

In the current study, we used bioinformatics to screen out the differentially expressed mRNAs and miRNAs in liver tissues of NASH patients, and verified the effect of miR-142-5p on TSLP and JAK-STAT signaling pathway and its role in NASH by constructing a NASH mice model, in order to find a new target for the treatment of NASH.

## RESULTS

### Bioinformatics analyses identifies target molecules

**Figure 1:**
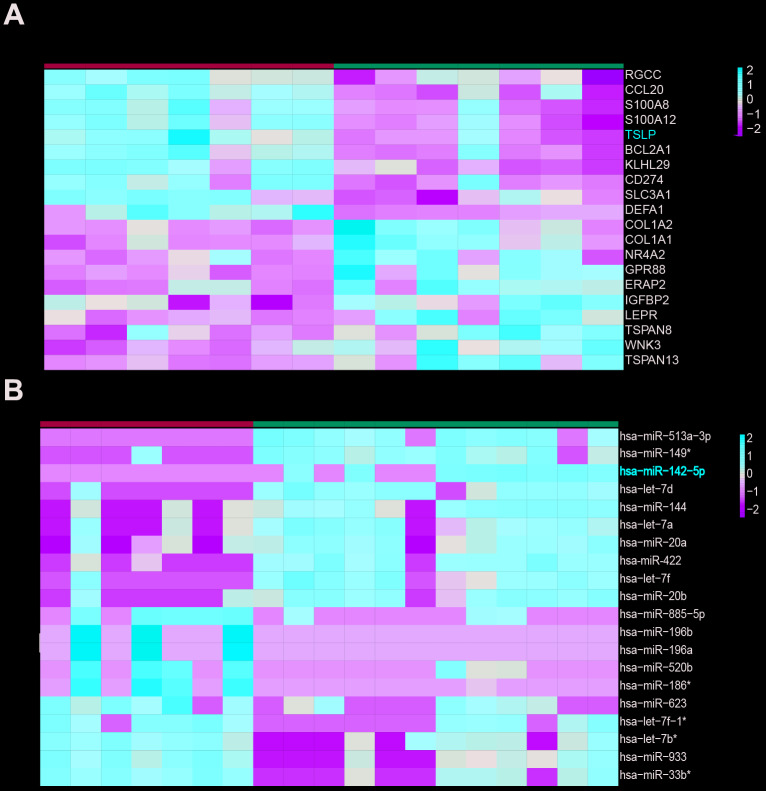
**TSLP was up-regulated and miR-142-5p was downregulated in NASH.** (**A**) Heatmap showing differentially expressed genes between health and NASH group. (**B**) Heatmap showing differentially expressed miRNAs between health and NASH group. The expression fold in NASH groups was calculated compared with the health groups. Log_2_|FC|>1 and adj. P < 0.05 was considered statistically significant.

**Figure 2:**
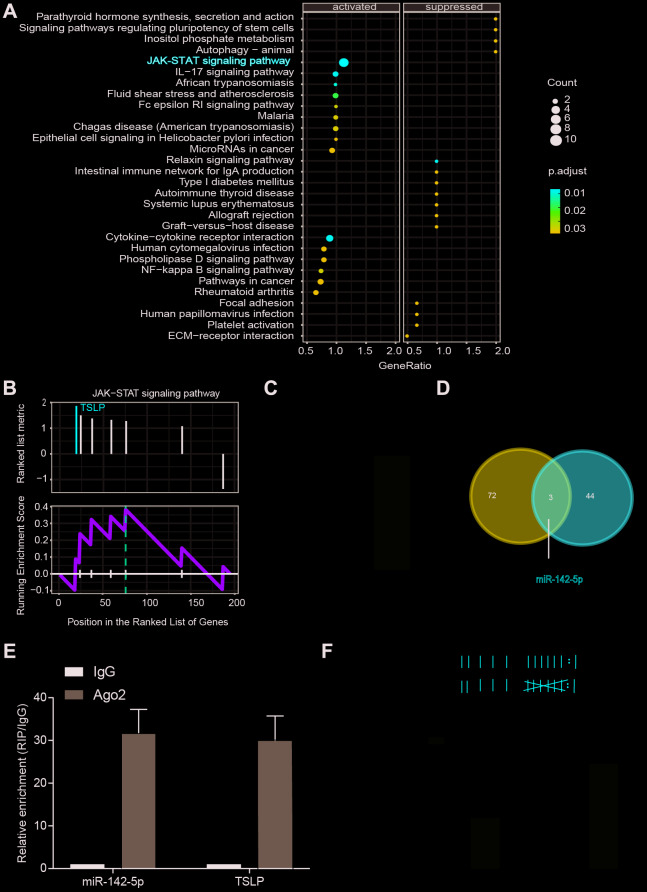
**JAK-STAT pathway was actived in NASH patients and verification of the target gene.** (**A**) Dotplot suggest the distributions of some biological pathway gene sets. The size of the circle represents the count value. The gradient changes of color represented adjust P values. (**B**) KEGG analysis of JAK-STAT pathway in NASH. (**C**) Expression of *TSLP* in NASH disease model. ^*^P < 0.05, compared with control group or WT group. (**D**) Venn diagram showing the overlap between dysregulated miRNAs and miRNAs that target at *TSLP*. (**E**) RIP assays of the enrichment of Ago2 on TSLP and miR-142-5p relative to IgG in liver. ^**^P < 0.01, compared with IgG group. (**F**) The miR-142-5p binding sites on TSLP were predicted by bioinformatics. TSLP wild-type form (TSLP-wt) and mutated form (TSLP-mut) were displayed on the left panel. Dual-luciferase reporter assay was conducted to identify the target relationship between miR-142-5p and TSLP. All data were means ± SD.

Microarray datasets of GSE63067 and GSE33857 from GEO database were analyzed to screen out the differentially expressed mRNAs and miRNAs, respectively. Heatmap reflected the top ten differentially expressed miRNAs and mRNAs. MiR-142-5p was downregulated and TSLP was upregulated in NASH (Figure 1). JAK-STAT signaling pathway was predicted to be activated in NASH (*P*=0.009, FDR=0.012) (Figure 2A, 2B). From the differential mRNAs combined with the literature, we selected *TSLP* for further research. The expression of *TSLP* in the NASH group and the control group was verified by Real-time PCR. The expression of *TSLP* in the NASH group was significantly increased (*P<0.05*) (Figure 2C). Furthermore, this study predicted the possible target miRNAs of *TSLP* by miRanda and found that miR-326, miR-142-5p and miR-331-3p were the same part of the difference results by the intersection of Venn diagram and differentially expressed miRNA (Figure 2D). RIP assays were performed utilizing the anti-Ago2 (the core component of the RISC) antibody. The results showed that TSLP and miR-142-5p are drastically enriched in Ago2 immunoprecipitates compared with those in the IgG (*P<*0.01) (Figure 2E), suggesting that TSLP physically existed in Ago2-based miRNA-induced repression complex and is associated with miR-142-5p (Figure 2E). We verified the three miRNAs by Real-time PCR. Compared with the control group, only miR-142-5p was significantly decreased and the difference was statistically significant (*P<0.05*) (Supplementary Figure 1). Dual-luciferase reporter assay was conducted to verify the target relationship between miR-142-5p and TSLP. TSLP-wt group showed the decreased luciferase activity versus TSLP-mut group (Figure 2F).

### Function of miR-142-5p to TSLP in the liver of NASH mouse model

**Figure 3:**
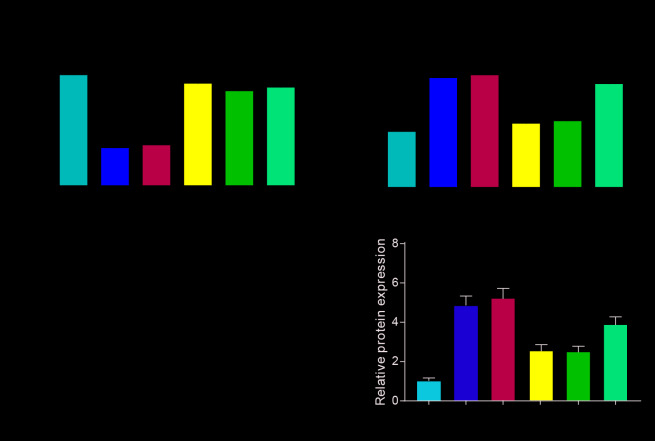
**The expression of miR-142-5p and TSLP and the function of miR-142-5p to TSLP in the liver of mouse model.** (**A**) The relative expression of miR-142-5p in the liver of different group. (**B**) Relative mRNA expression of TSLP in the liver of different mouse group. (**C**) Protein expression of TSLP in the liver of different mouse group; WT group: wild-type mice fed a chow diet (CD); blank group: Ldlr−/− mice fed an high-fat diet (HFD) treated with phosphate buffer saline (PBS); NC group: Ldlr−/− mice fed an HFD treated with control agonis; A142 group: Ldlr−/− mice fed an HFD treated with agonist of miR-142-5p; A142-C group: Ldlr−/− mice fed an HFD treated with miR-142-5p agonist and JAK-STAT signaling pathway activator-colivelin; A142-TSLP group: Ldlr−/− mice fed an HFD treated with miR-142-5p agonist and AAV/TSLP. AAV: adeno-associated virus. n=6. ^*^P < 0.05; ^**^P < 0.01, compared with WT group; ^#^ *P*< 0.05; ^##^*P* < 0.01, compared with NC group; ^^^*P* < 0.05, compared with A142 group. All data were means ± SD.

The miR-142-5p expression was more markedly increased by mir-142 (16 mg/kg) than mir-142 (8 mg/kg) in the preliminary experiments (data not shown). Since, the mice in the A142 group were treated with 16 mg/kg mir-142. Wild-type C57BL/6 mice untreated (WT group) were used as control. Based on the established NASH mouse model, the expression of miR-142-5p and *TSLP* were evaluated. The Ldlr-/- mice fed with HFD (blank group) presented a decreased expression level of miR-142-5p compared with WT mice under CD (WT group). In NASH model, the mice treated with miR-142-5p agonist (A142 group) showed an upregulated expression of miR-142-5p compared with NC group (*P*<0.01) (Figure 3A). At the same time, we detected the TSLP expression based on the same grouping. TSLP was upregulated in the NASH mouse model compared with WT mice under CD. When the NASH mice treated with miR-142-5p, the TSLP was downregulated versus the NASH mice treated with mir control. The NASH group treated with miR-142-5p-colivelin could also down regulate the expression level of TSLP (Figure 3B). In accordance with western blot results, the protein expression of TSLP was increased in the model group and miR-142-5p downregulated the TSLP expression. Besides, AAV/TSLP could upregulate the TSLP expression, which was reversed by miR-142-5p (Figure 3C).

### JAK-STAT signaling pathway in the NASH mouse model

**Figure 4:**
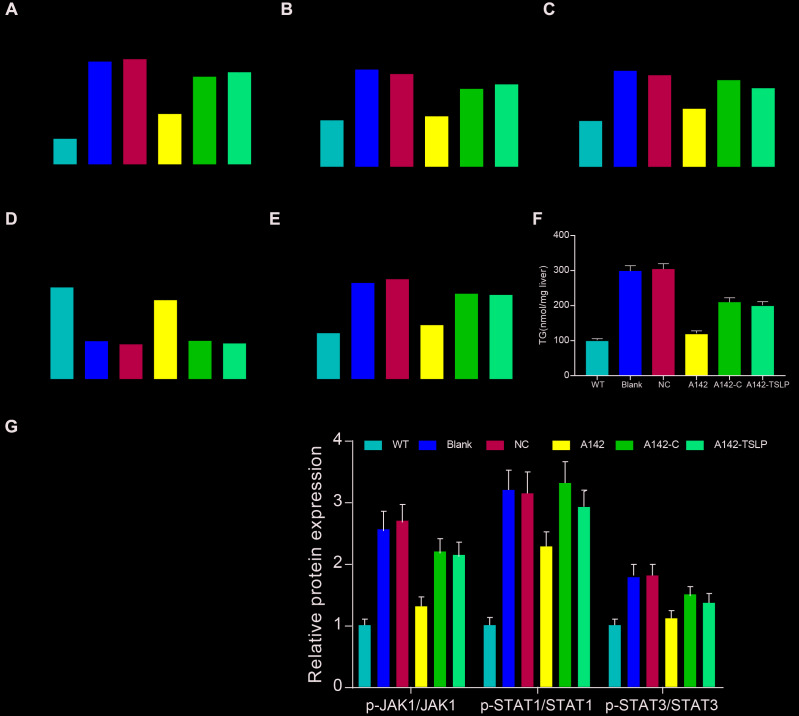
**The expression of inflammatory factors and JAK-STAT signaling pathway biomarkers in the NASH mouse model.** (**A**) The mRNA expression level of tumor necrosis factor (TNF)-α. (**B**) The mRNA expression level of interferon (IFN)-β. (**C**) The mRNA expression level of liver monocyte chemoattractant protein-1 (MCP1). (**D**) The mRNA expression level of IL-4. (**E**) The mRNA expression level of IL-6. (**F**) The expression level of TG in the liver. (**G**) The protein expression of the biomarkers of JAK-STAT signaling pathway. All data were means ± SD. ^*^P < 0.05, compared with WT group; ^#^*P* < 0.05, compared with NC group; ^^^*P* < 0.05, compared with A142 group.

TNF-α, IFN-β, IL-6 and MCP-1, the pro-inflammatory factors were overexpressed in the Ldlr-/- mice fed with HFD compared with WT mice fed with CD. The anti-inflammatory factor IL-4 were decreased in the blank group compared with WT group. MiR-142-5p played an inhibiting inflammation role in NASH model. The expression of TNF-α, IFN-β, IL-6 and MCP-1 was all upregulated and IL-4 expression were downregulated in the group treated with miR-142-5p versus the NC group, and it could be rescued by colivelin or AAV/TSLP (Figure 4A–4E). In addition, we tested the TG content and found that the A142 group can significantly reduce the TG content (*P*<0.05). However, this therapeutic effect was reversed by the agonist group and the overexpressed TSLP group (Figure 4F). Western blot was conducted to detect whether JAK-STAT signaling pathway was activated in the NASH group. The data showed that JAK-STAT pathway related biomarkers were upregulated in the NASH model compared with WT group (Figure 4G, *P* < 0.05). Besides, miR-142-5p treatment group significantly inhibited the phosphorylation of the JAK-STAT signaling pathway. The use of activators and overexpressing TSLP groups reversed this inhibition. (Figure 4G, *P* < 0.05).

### MiR-142-5p alleviates NASH through TSLP and JAK-STAT signaling pathway

**Figure 5:**
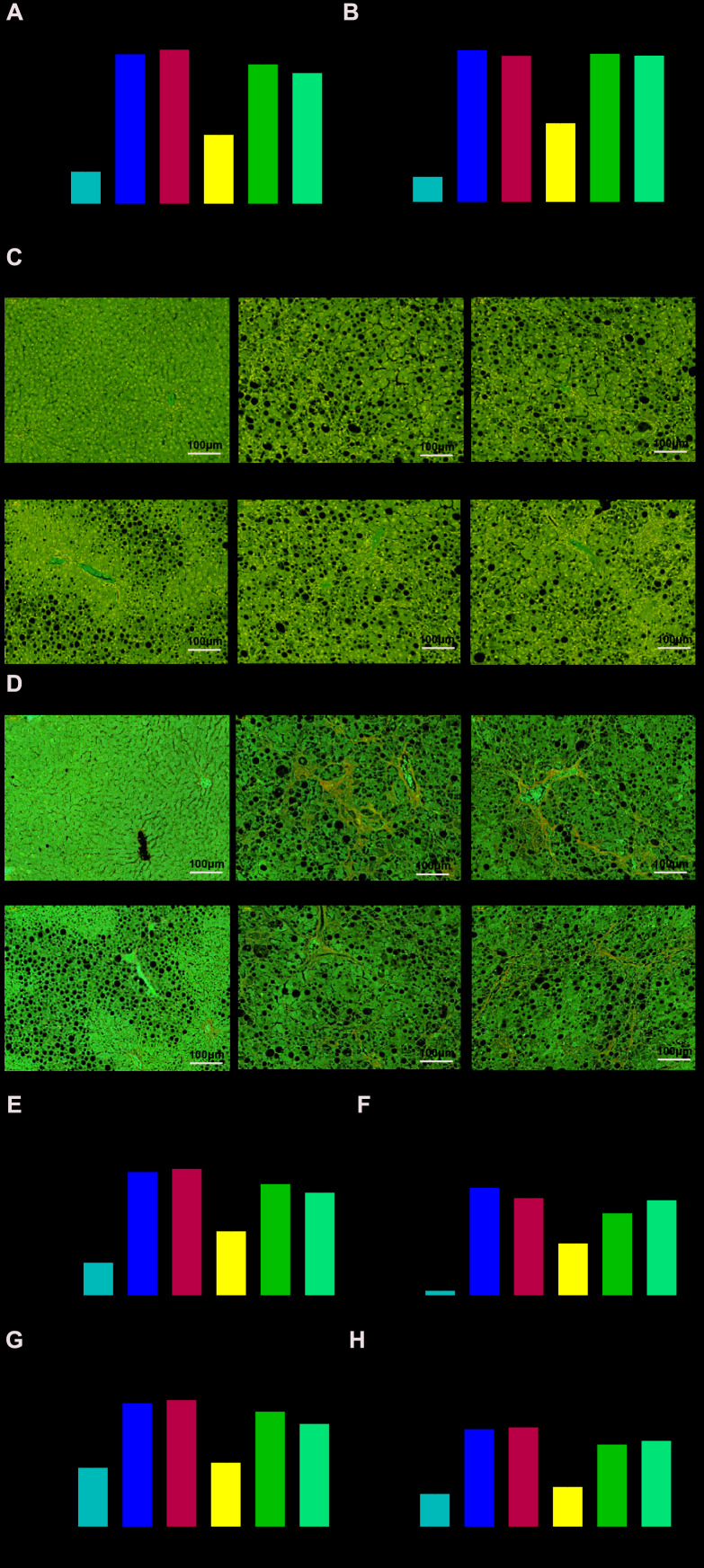
**miR-142-5p reduced liver injury, liver fatty degeneration and liver fibrosis, which could be rescued by JAK-STAT signaling pathway activator or AAV/TSLP.** (**A**) The level of alanine aminotransferase (ALT) secreted into serum. (**B**) The level of aspartate aminotransferase (AST) secreted into serum. (**C**, **E**) H&E staining for liver fatty degeneration. Original magnification ×200. (**D**, **F**) The representative images of collagen deposition in Masson’s trichrome. The expression of fibrosis-related genes. Original magnification ×200. (**G**) The mRNA expression level of Tgfβ and (**H**) The mRNA expression level of Collagen-1 α2. ^*^P < 0.05, compared with WT group; ^#^*P* < 0.05, compared with NC group; ^^^*P* < 0.05, compared with A142 group. All data were means ± SD.

Serum ALT and AST secretion were both markedly increased in NASH group compared with WT group (Figure 5A, 5B). Histological examination of liver fatty degeneration and liver fibrosis gave further evidence that phenomenon of hepatocyte fat accumulation and collagen deposition could be seen in NASH model (Figure 5C–5F). Further, liver expression of TGF-β and collagen-1 α2 (fibrosis-related genes) was increased in NASH group compared with WT group (Figure 5G, 5H). All these signs suggested the mouse model of NASH has been constructed successfully. Based on the NASH mouse model, the NASH mice treated with miR-142-5p had the lower serum level of ALT and AST compared with NC group; the NASH mice treated with miR-142-5p and colivelin or miR-142-5p and AAV/TSLP performed the higher level of AST and ALT compared with NASH mice treated with miR-142-5p (Figure 5A, 5B). Alleviation role of miR-142-5p could also be validated via assessments of hepatic tissue alteration in which liver performed fatty degeneration and liver fibrosis compared with NC group. The above hepatic tissue alteration caused by miR-142-5p could be reversed by JAK-STAT signaling pathway activator colivelin or AAV/TSLP (Figure 5C–5F). Besides, we investigated the liver fibrosis on gene level and the over expressed TGF-β and collagen-1α2 were seen in miR-142-5p group compared with NC group, indicating miR-142-5p ago could protect liver fibrosis against NASH. To investigate whether miR-142-5p inhibit NASH fibrosis through JAK-STAT signaling pathway or *TSLP*, we detected the TGF-β and collagen-1α2 expression in miR-142-5p-colivelin group and miR-142-5p-TSLP group. The results revealed that TGF-β and collagen-1α2 expression was increased in A142-c group and A142-TSLP group compared with A142 group (Figure 5G, 5H).

### Effect of molecular interference sequence on miR-142-5p and TSLP

**Figure 6:**
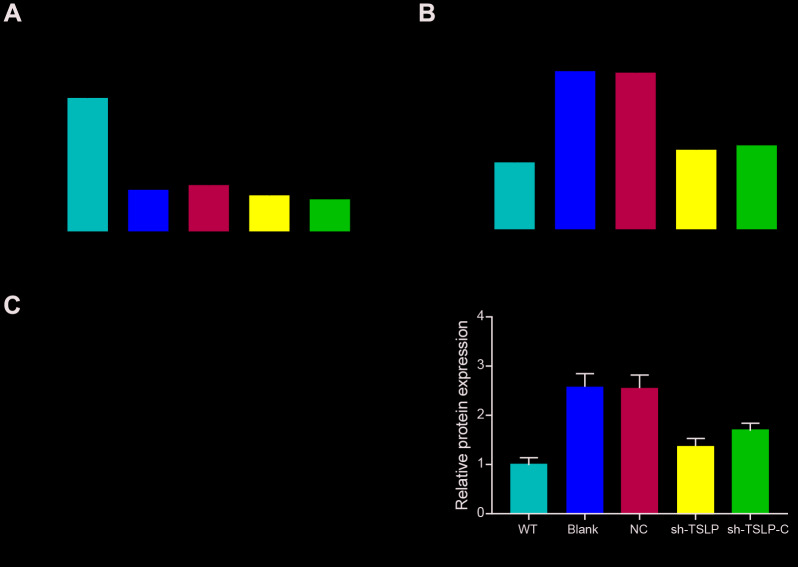
**The effect of molecular interference sequences on the expression of miR-142-5p and TSLP.** (**A**) Relative expression level of miR-142-5p in different groups. (**B**) The relative mRNA expression of TSLP. (**C**) The protein expression level of TSLP. WT group: wild-type mice fed a chow diet; blank group: Ldlr−/− mice fed an HFD treated with phosphate buffer saline; NC group: Ldlr−/− mice fed an HFD treated with sh-control; sh-TSLP group: Ldlr−/− mice fed an HFD treated with sh-TSLP; sh-TSLP-C group: Ldlr−/− mice fed an HFD treated with sh-TSLP and colivelin (JAK-STAT signaling pathway activator). Each group contained 6 mice. ^*^P < 0.05, ^**^P < 0.01, compared with WT group; ^#^*P* < 0.05, compared with NC group. All data were means ± SD.

MiR-142-5p expression was decreased and *TSLP* expression was increased in the Ldlr-/- mice fed with HFD (blank group) in comparison with WT mice fed with CD (WT group). Besides, knockdown *TSLP* made no change to miR-142-5p expression but exhibited the inhibited expression of *TSLP* compared with NC group. Further, we investigated whether colivelin could influence the miR-142-5p or *TSLP* expression. The results showed that there was no relationship between colivelin and the expression of miR-142-5p and TSLP (Figure 6).

### Effect of TSLP in the NASH mouse model through JAK-STAT signaling pathway

**Figure 7:**
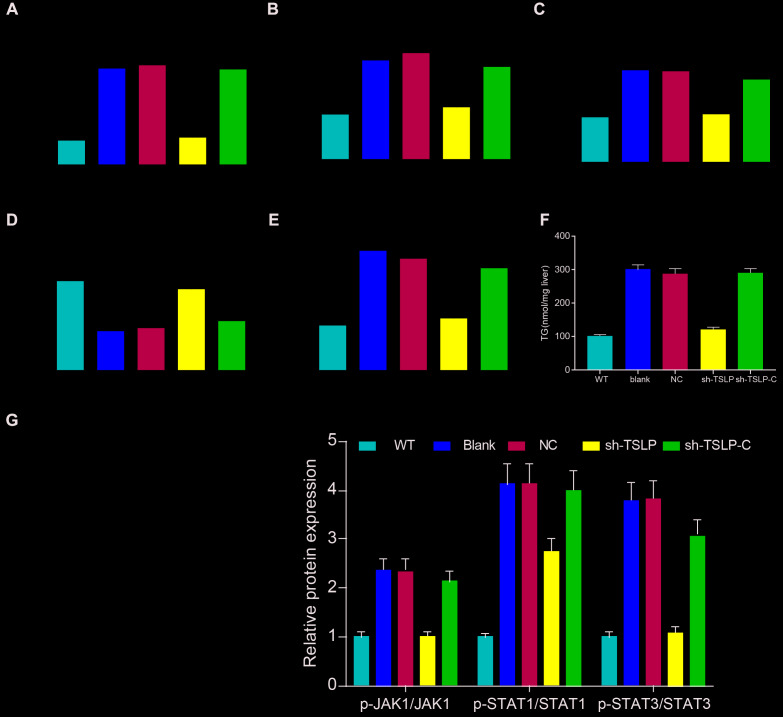
**Downregulating TSLP inhibited the expression of inflammatory factors and JAK-STAT pathway biomarkers, which could be rescued by JAK-STAT signaling pathway activator.** (**A**) The mRNA expression level of tumor necrosis factor (TNF)-α. (**B**) The mRNA expression level of interferon (IFN)-β. (**C**) The mRNA expression level of liver monocyte chemoattractant protein-1 (MCP1). (**D**) The mRNA expression level of IL-4. (**E**) The mRNA expression level of IL-6. (**F**) The expression level of TG in the liver. (**G**) The protein expression of the biomarkers of JAK-STAT signaling pathway. ^*^P < 0.05; ^**^P < 0.01, compared with WT group; ^#^*P* < 0.05; ^##^*P* < 0.01, compared with NC group; ^&^*P* < 0.05; ^&&^*P* < 0.01, compared with sh-TSLP group. All data were means ± SD.

The expression of TNF-α, IFN-β, IL-6 and MCP-1 was over expressed and the expression of IL-4 was down regulated in NASH mouse model, indicating the high level of inflammation in NASH. Knockdown TSLP in NASH mice generally inhibited the pro-inflammatory factors expression levels and increased the anti-inflammatory factors expression, which suggested the inflammatory acceleration of TSLP in NASH. In addition, pro-inflammatory factors were upregulated and anti-inflammatory factors was down regulated in the NASH mice treated with AAV/sh-TSLP and colivelin compared with NASH mice only treated with AAV/sh-TSLP (Figure 7A–7E). In addition, we examined TG and found that knockdown of the TSLP group significantly reduced TG expression (*P*<0.05), but the pathway activator group was able to reverse this therapeutic effect (Figure 7F). Further, as shown in the (Figure 7G), p-JAK1, p-STAT1 and p-STAT3 were decreased in the sh-TSLP group (*P*<0.05). This result indicated that JAK-STAT pathway was activated in the Ldlr-/- mice fed with HDF, suppressed in the NASH mice treated with AAV/sh-TSLP compared with NASH mice treated with sh-TSLP control and activated in the NASH mice treated with AAV/sh-TSLP and colivelin compared with sh-TSLP group.

### Knockdown TSLP reduced liver injury, fatty degeneration and fibrosis

**Figure 8:**
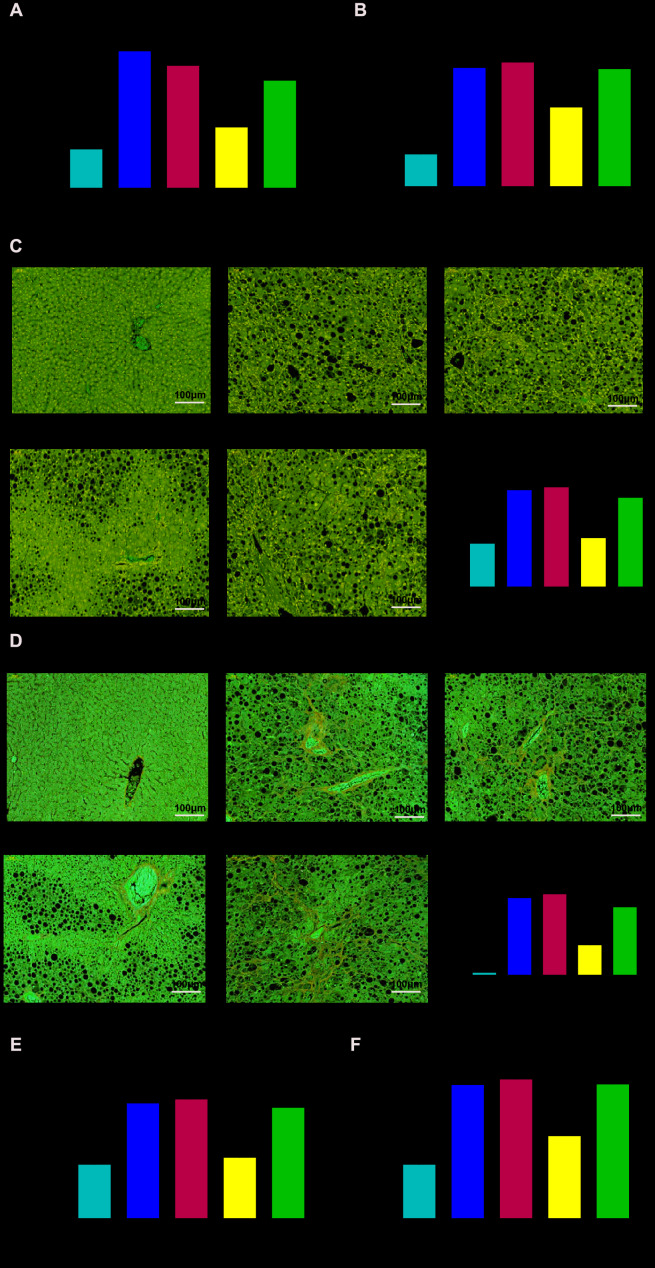
**Knockdown TSLP reduced liver injury, liver fatty degeneration and liver fibrosis, which could be rescued by JAK-STAT signaling pathway activator.** (**A**) The level of alanine aminotransferase (ALT) secreted into serum. (**B**) The level of aspartate aminotransferase (AST) secreted into serum. (**C**) Liver fatty degeneration was detected by H&E staining; (**D**) The degree of fibrosis was shown by Masson’s trichrome Original magnification ×200. (**E**) The mRNA expression level of Tgfβ and (**F**) The mRNA expression level of Collagen-1α2. ^*^P < 0.05, compared with WT group; ^#^*P* < 0.05, compared with NC group; ^&^*P* < 0.05, compared with sh-TSLP group. All data were mean ± SD.

As expected in the NASH model, the serum ALT and AST level, liver fatty degeneration, liver fibrosis and fibrosis-related genes expression were increased in the Ldlr-/- mice with HFD compared with WT mice with CD. As for the function of TSLP in NASH, we detected the above indicators and found that ALT and AST were secreted less, the fatty degeneration degree and fibrosis degree of liver tissue were attenuated and fibrosis-related gene expression were downregulated in the NASH mice treated with AAV/sh-TSLP compared with NC group. When we added colivelin into sh-TSLP group, the indicators as for NASH has been reversed compared with sh-TSLP group (Figure 8).

## DISCUSSION

NASH is a serious liver disease which can be defined by necro-inflammation, a variable amount of fibrosis and potential for progression to cirrhosis [7]. Recent evidence suggests that serum levels of specific miRNAs may vary significantly in patients with NAFLD/NASH, depending on the stage of the disease. Clearly, the role and correlation of the target miRNA needs to be thoroughly examined in various in vivo models and with different approaches before clinical trials of miRNA-based therapies can be conducted [20, 21]. However, the lack of available biomarkers to diagnose different stages of NAFLD with a non-invasive strategy is one of the greatest challenges facing clinicians today [22]. Therefore, in this study, the expression of miR-142-5p in NASH and its therapeutic effect were explored through the NASH mouse model. Our study demonstrated that miR-142-5p can reduce liver inflammation and fibrosis by targeting TSLP and inhibiting the JAK-STAT pathway.

MiRNA reveals a biological function by affecting the expression of a target gene by incompletely binding to a target gene [23]. MiRNAs are highly conserved and specific in expression, so they have small differences among different species and have better molecular markers for disease diagnosis and targeted therapy [24]. Although no clinical trials have been designed to test miRNA-based therapies for NAFLD/NASH. However, the use of pharmacological inhibitors of miR-122 and miR-34 in other liver diseases has therapeutic implications. MiR-122 antisense LNA (Miravelsson) is currently in phase II clinical trials and has been shown to reduce viral RNA load in HCV infected patients without causing viral resistance [25, 26]. In addition, another clinical trial established in compound MRX34 showed an effective downregulation of miR-34 targeting cancer promoters, but this clinical trial required the cessation of severe immune-related adverse events [27]. The failure of this and other experimental evidence suggests that in order to improve the efficiency and safety of miRNA-based therapies, two issues need to be addressed: improving the specific delivery of miRNA drugs to target organs and preventing off-target effects in healthy organs. These important issues are part of the next challenge that clinicians and scientists need to face to address the development of miRNA-based therapies for NAFLD/NASH and other diseases [28].

Currently, high-throughput technology has been identified as an effective means of detecting miRNAs that may be involved in the development of disease or have therapeutic potential in tissues or blood circulation disorders. In addition, luciferase reporter assays are gold markers for in vivo confirmation of pathologic miRNA/mRNA interactions [22, 29]. Therefore, this study used the GEO database to pre-screen the differential expressions of mRNAs and miRNAs in NASH. TSLP and miR-149-5p were revealed to be differentially expressed in NASH. Studies have shown that *TSLP* encodes a hemopoietic cytokine and promotes T helper type 2 cells responses, which are linked to immune response in various inflammatory diseases, including asthma, allergic inflammation and chronic obstructive pulmonary disease. Li et al. found that *TSLP* promoted MRC-5 cells fibrosis and activated the expression of MAPK7, p-p38, p-ERK1 and p-JNK1 which are the biomarkers of MAPK pathway. The part of *TSLP* in MRC-5 cells indicated the potential therapeutic implication of *TSLP* in lung fibrosis of acute lung injury [30]. Sansonno et al. found that *TSLP* was significantly overexpressed in liver tissue and serum of hepatitis C virus-related cryoglobulinemic vasculitis [31]. Lee et al. investigated the substantial levels of hepatocyte *TSLP* in fibrotic liver tissue from chronic hepatitis C virus patients and detected the high level of *TSLP* expression [32]. Despite the important role of *TSLP* as regulator of inflammation and fibrosis in many diseases, the function of *TSLP* in NASH remains enigmatic. In this study, its high expression in NASH disease was also detected in the validation of animal models of disease. In addition, we further predicted the miRNAs regulated TSLP by miRanda and selected the miRNAs of this study by the selection of the miRNAs differentially expressed with the GEO database. MiR-326, miR-142-5p and miR-331-3p were the results after the intersection. Subsequently, only miR-142-5p was found to be statistically different in the detection of the three miRNAs in the NASH mouse model. Further dual luciferase reporter experiment revealed that the target gene of miR-142-5p was *TSLP*, so we believed that miR-142-5p might play a role in the occurrence of NASH by affecting the expression of *TSLP*. Similarly, a previous study showed that miR-142-5p can affect the expression of fibrotic genes, and fibrosis is one of the important causes of NASH [9]. Therefore, this study further validates it, but the mechanism that leads to NASH still needs further study.

The KEGG analysis of NASH further showed that the JAK-STAT signaling pathway may play a role in NASH. This pathway have been elucidated to play a vital role in the signal transduction in the pathogenesis of many diseases [33]. Accumulating evidences suggested that JAK-STAT signaling pathway was involved in the development of inflammatory diseases. For example, Nicolas et al. reviewed that dysregulation of JAK-STAT pathway could be found in inflammation, cancer and neurodegenerative diseases. Similarly, Cai et al. suggested that JAK/STAT pathway has been shown related to the release of various cytokines and inflammatory mediators and involved in the regulation of immune response in sepsis [34]. At the same time, the role of JAK-STAT in NASH was verified by recent studies [35]. In addition, Wohlmann et al. found that *TSLP* triggers inflammatory responses in the course of atopic diseases through JAK-STAT signaling pathway [36]. Consistent with previous studies, the effects of miR-142-5p and *TSLP* on NASH and their effects on JAK-STAT signaling pathway were verified by establishing animal models of overexpression and down-regulation of miR-142-5p and *TSLP*, respectively.

ALT and AST are one of the important indicators reflecting the normal function of liver tissue, and its elevated content is often an important marker of liver function damage [37]. TNF-α, IFN-β, IL-6, MCP-1 and IL-4 are important inflammatory or anti-inflammatory factors, which are also one of the indicators of liver tissue damage [38, 39]. In this study, the morphological observation combined with biochemical indicators to verify the extent of miR-142-5p and *TSLP* damage to NASH and whether it produced therapeutic effects. The results showed that overexpression of miR-142-5p resulted in a decrease in the expression of *TSLP* and a certain degree of reversal of liver damage caused by NASH, and the therapeutic effect of the experimental group after additional addition of *TSLP* was attenuated or even disappeared. Similarly, from the results, the therapeutic effect on NASH was significantly reduced by down-regulating the expression of *TSLP*. In the validation of the expression of JAK-STAT signaling pathway, we can see that when the expression level of miR-142-5p is increased, the expression of *TSLP* is decreased, and the expression of phosphorylated protein is decreased when the total protein expression is unchanged, thereby the activity of the JAK-STAT signaling pathway is inhibited. Consistent with our findings, it is well known that when the total protein is unchanged, the phosphorylation level of the corresponding protein is decreased or the phosphorylation level is decreased, the activity of inhibiting the signaling pathway is produced [40]. In addition, we have established a group of miR-142-5p and JAK-STAT signaling pathway activators to further validate their effects on NASH. The results show that the addition of a signaling pathway activator after over expression of miR-142-5p leads to its therapeutic effect of NASH is weakened. Conversely, when the expression of *TSLP* is elevated, the JAK-STAT signaling pathway is activated, further aggravating the degree of liver damage in NASH. Therefore, we have reason to believe that miR-142-5p may affect the occurrence and development of NASH by regulating the expression of *TSLP* and then affecting JAK-STAT signaling pathway. This result is consistent with previous studies examining the relationship between JAK-STAT signaling pathway and NASH [17].

In summary, our study first proposed that miR-142-5p might be a protect factor in NASH. It could inhibit progression of NASH via TSLP/JAK/STAT axis, which provided a new potential therapeutic target of NASH. However, the limitation of this study lies in the fact that this part of the results is only proved by animal models and not in-depth discussion of its biological effect targets in combination with clinical investigation. Therefore, it is important to carry out appropriate clinical sample verification and in-depth mechanism exploration, and to provide evidence for the treatment of NASH through continuous in-depth research.

## MATERIALS AND METHODS

### Bioinformatics analysis

Microarray datasets GSE63067 and GSE33857 were obtained from Gene Expression Omnibus (GEO) database (http://www.ncbi.nlm.nih.gov/geo/). The dataset of GSE63067 was analyzed to visualize differentially expressed mRNAs, from which 7 human non-alcoholic steatohepatitis (NASH) and 7 healthy human liver tissues were chosen. The dataset of GSE33857 was used for aberrant expressed miRNAs. Differentially expressed genes (DEGs) as well as dysregulated pathways were uncovered through R packages and KEGG database. The target relationship between miRNA and mRNA was predicted by TargetScan 7.1 (http://www.targetscan.org/vert_71/). Differentially expressed mRNAs and miRNAs were screened out for further analysis with the criteria of |log_2_FC| > 1 and adj. P < 0.05 by Empirical Bayes method. The clustering analysis was based on “affy”, “affyPLM”, “RColorBrewer”, and “heatmap” packages.

### AAV vector construction

To generate AAV/TSLP or AAV/sh-TSLP, the mouse TSLP cDNAs (Genepharma, China) were ligated downstream from the cytomegalovirus immediate early promoter within the gutted adeno-associated virus-2 (AAV) vector. We chose the CMV promoter for overexpression of TSLP plasmid (GV410, CMV bGlobin-MCS-3FLAG-T2A-EGFP) was constructed by Gene Chem Co. (Shanghai, China). While, a plasmid with U6 as a promoter was selected to interfere with the expression of TSLP (GV478, U6-MCS-CAG-EGFP) was constructed by Gene Chem Co. (Shanghai, China).

### NASH mouse models

Animal-based experiments performed in this experiment were approved by Sichuan Academy of Medical Sciences and Sichuan Provincial People’s Hospital animal experiment ethics committee. The mice were all received human care according to the Guide for the Care and Use of Laboratory Animals (National Institutes of Health). According to the predecessor’s NASH modeling method [13], all operation was conducted on C57BL/6 mice. Mice in the WT group were fed with chow diet (CD, n=18). Models of NASH were conducted by eight-week-old Ldlr−/− mice (with the same genetic background, C57BL/6) fed with high fat diet (HFD, n=18) for 14 weeks, which were purchased from Charles River (MA, USA). HFD contained 15% cacao butter and 1.25% cholesterol but no choline chloride. At the age of 8 weeks old, the mice models were injected with AAV/NEO (control group), AAV/TSLP, or AAV/sh-TSLP virus, and each group had 6 mice. When the mice were 9 to 10 weeks old, they were injected with other two booster injections. Per mouse was injected with 200 μL virus at a titer of 1 × 10^10^ PFU of each vector through tail intravenous injections. At last genomic copies of AAV (2 × 10^10^ to 2 × 10^11^) were used for tail-vein injections. Colivelin (MedChem Express, NJ, USA), a neuroprotective peptide and activator of STAT3, was used as an activator of JAK/STAT3 signaling pathway. At the age of 12 to 17 weeks old, mice were treated with 16 mg/kg mir-142, mir control, or PBS through tail intravenous injections (once a day for three days). The mir-142 and mir control were purchased from Genepharma (Genepharma, China). To activate JAK-STAT signaling pathway, mice were given 20 μg colivelin in 0.5ml physiological saline intraperitoneally for continuous 6 days.

All mice were sacrificed at 22 weeks by sedating them with 2.5% isofluorane. Mice blood was collected from inferior vena cava, centrifuged at 1500 g for 15 min, and stored it in −80°C. After that, livers were isolated and froze in liquid nitrogen quickly. Finally, we fixed the tissue in formalin and paraffin-embedded or stored it in −80°C.

### RNA isolation and expression analysis

Trizol reagent (Beyotime, Shanghai, China) were applied to extract the all the RNA of the cell or tissue fragment, then PrimeScript RT Reagent Kit (Takara, Tokyo, Japan) was employed for synthesis of cDNA under the instruction from manufacture. The miRNA was converted to cDNA using miScript cDNA synthesis kit (Qiagen, Dusseldorf, Germany) following the manufacturer’s protocol. Later, SYBR Select Master Mix and ABI Prism 7000 Sequence Detection System (Applied Biosystems, Foster City, CA, USA) were applied to carrying out qRT-PCR. β-actin and U6 were chosen as internal references for mRNA and miRNAs respectively. Relative expressions of mRNAs and miRNAs were quantified using 2^^-ΔΔCt^ method. Supplementary Table 1 displayed all the primer sequences.

### Dual luciferase reporter experiment

A 3’-UTR wild type (wt) or mutant 3’-UTR (mut) of TSLP were constructed by PCR, and were then inserted into the multiple cloning sites in the pRL-TK Vector (Promega, WI, USA). Human 293T cells were cultured in six-well plate at the confluence of 70-80% and then were co-transfected with pRL-TK-TSLP-3’-UTR mut or pRL-TK-TSLP 3’-UTR wt plasmid in combination with 100 nM miR-142-5p or 100 mM negative control, respectively. Lipofectamine 2000 purchased from Invitrogen was used to transfect the vector. The luciferase activities after 48 h co-transfection were assessed by a Dual-Luciferase® Reporter assay system (Promega) and were normalized to firefly luciferase activity.

### RNA immunoprecipitation assays

RNA immunoprecipitation (RIP) assays were implemented using the Magna RIP RNA-binding Protein Immunoprecipitation Kit (Millipore), according to the manufacturer’s instructions. Anti-Ago2 antibody and normal IgG (Millipore) were used for immunoprecipitation. The co-precipitated RNAs were purified with phenol: chloroform: isoamyl alcohol and subsequently analyzed by qPCR to assess the enrichment of miR-142-5p and TSLP to Ago2.

### Histological analysis

3 μm thick paraffin-embedded liver sections were stained with hematoxylin and eosin (H&E) and Masson’s trichrome to evaluate hepatic steatosis, inflammation, and liver fibrosis using an Olympus microscope (Tokyo, Japan). Hematoxylin and eosin staining were used for NAFLD activity score evaluation (NAS) [41]. Masson’s trichrome to determine the collagen distribution. Evaluation using the methods described by the predecessors [42].

### Metabolic analyses

Olympus AU5400 automatic chemical analyzer (Olympus) was conducted to detect the levels of serum alanine aminotransferase (ALT) and aspartate aminotransferase (AST), which were reliable indicators of liver inflammatory injury, following the manufacturer’s instructions.

**Triglyceride assay**

Triglyceride (TG) levels were measured by a TG kit (Nanjing, China). The values obtained were normalized to total protein concentrations. Protein concentrations were measured using the BCA method.

### Western blot

Frozen mouse liver tissue samples (about 50 mg) were homogenized in 650 mL of TNE buffer (1 mmol/L EDTA, 1% Nonidet P40, 10 mmol/L Tris-HCl (pH 7.5), 150 mmol/L NaCl) containing a phosphatase inhibitors and cocktail of protease (Roche, Basel, Switzerland). The incubation condition was 4°C for 45 min and the centrifugation condition was at 12 000 g for 5 min. BCA Protein Assay (Thermo Fisher, MA, USA) was conducted to quantified protein content was quantified in the supernatant. Reducing sample buffer (Bio-Rad) was used to mix lysates for electrophoresis and subsequently the lysates were transferred onto polyvinylidene fluoride membranes (Thermo Fisher). Equal loading (50 mg) was verified using Ponceau red solution. Antibodies against TSLP (ab188766) was purchased from Abcam (MA, USA) and p-JAK1 (#3331S), JAK1 (#50994S), p-STAT1 (#9167S), STAT1 (#14994S), p-STAT3 (#52075S), STAT3 (#9139S) and anti-β-actin (#3700S) were obtained from Cell Signaling Technology (Danvers, MA, USA). After incubating with HRP-labeled goat anti-rabbit IgG (H+L) (1:3000; Beyotime, Shanghai, China), the Clarity Western ECl Substrate (BioRad, CA, USA) was conducted to proceed immunodetection and the Las-4000 Imaging System and Image MultiGauge software (Fujifilm, Tokyo, Japan) were used to reveal bands.

### Statistics

All the statistical analyses were performed by GraphPad Prism (vesion6.0) software. The form of means ± standard deviation (SD) was used for the whole results. Student t-test (unpaired t-test) was used to compare means between differentiated and undifferentiated groups. Differences among the three groups were analyzed via one-way analysis of variance (ANOVA), Tukey’s test was performed in all pairwise comparisons. P values < 0.05 were considered as statistically significant.

### Ethical approval

All procedures performed in studies involving animals were in accordance with the ethical standards of the Sichuan Academy of Medical Sciences and Sichuan Provincial People’s Hospital.

## Supplementary Material

Supplementary Figure 1

Supplementary Table 1
