# Supplementary material for: Overexpression of miR-142-5p inhibits the progression of nonalcoholic steatohepatitis by targeting TSLP and inhibiting JAK-STAT signaling pathway
Source: Aging (Albany NY). 2020 May 15;12(10):9066–84. doi: 10.18632/aging.103172 (PMC7288945; doi:10.18632/aging.103172)
Supplement: Supplementary Figure 1 [file aging-12-103172-s001..pdf]

SUPPLEMENTARY FIGURE

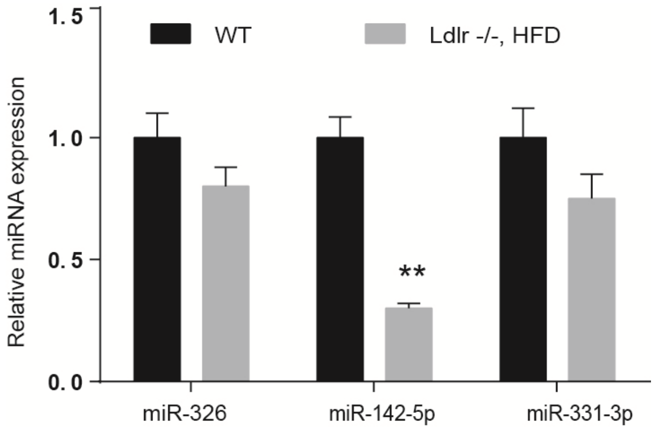

Supplementary Figure 1. The expression of three miRNAs in the NASH model. The expression of three miRNAs in the NASH model, **P* < 0.05, compared with WT group.
