# Supplementary material for: Overexpression of miR-142-5p inhibits the progression of nonalcoholic steatohepatitis by targeting TSLP and inhibiting JAK-STAT signaling pathway
Source: Aging (Albany NY). 2020 May 15;12(10):9066–84. doi: 10.18632/aging.103172 (PMC7288945; doi:10.18632/aging.103172)
Supplement: Supplementary Table 1 [file aging-12-103172-s002..pdf]

SUPPLEMENTARY TABLE

Supplementary Table 1. The primers sequences used for qRT-PCR.

PCR primers sequences	Forward (5′-3′)	Reverse (5′-3′)
miR-142-5p	CATAAAGTAGAAAGCACTACT	GCGAGCACAGAATTAATACGAC
miR-326	CCCAGGGCAGCAAACTCAGGACCAACTCCAAA	TTTGGAGTTGGTCCTGAGTTTGCTGCCCTGGG
miR-331-3p	TGCGGGCCCCTGGGCCTATC	CCAGTGCAGGGTCCGAGGT
TSLP	GATCAGGAAGACTCCACGTTCAGG	GTTTCAGGAGCCAGGAGAACAATCTG
TNF-α	CCCAGGCAGTCAGATCATCTTC	AGCTGCCCCTCAGCTTGA
IFN-β	GGATGCAGGAAGGAGATCACTG	CGATCCACACGGAGTACTTG
IL-4	CACAACTGAGAAGGAAACCTTCTG	CTCTCTCATGATCGTCTTTAGCCTTTC
IL-6	GGTACATCCTCGACGGCATCT	GTGCCTCTTTGCTGCTTTCAC
MCP-1	CCCCAGTCACCTGCTGTTAT	AGATCTCCTTGGCCACAATG
TGF-β	AAC AAT TCC TGG CGT TAC CTT	CTG CCG TAC AAC TCC AGT GA
collagen-1α2	CAGAACATCACCTACCACTGCAA	TTCAACATCGTTGGAACCCTG
β-actin	AGTGTGACGTTGACATCCGTA	GCCAGAGCAGTAATCTCCTTCT
